# Simultaneous Coexistence of Thyrotropin-Prolactin-Secreting Adenoma and Papillary Thyroid Carcinoma

**DOI:** 10.1155/2021/6564765

**Published:** 2021-11-30

**Authors:** Somaya Safi, Yousra Benabdelfedil, Sara Derrou, Faycal El Guendouz

**Affiliations:** ^1^Department of Endocrinology, Moulay Ismail Military Hospital, Meknes, Morocco; ^2^Department of Fundamental Sciences, Laboratory of Human Pathology Biomedicine and Environment, Faculty of Medicine and Pharmacy of Fez (FMPF), Sidi Mohammed Ben Abdellah University (USMBA), Fez, Morocco

## Abstract

**Background:**

The thyrotropin-secreting adenomas are very rare and even more rare when they simultaneously coexist with thyroid carcinoma. So far, only sixteen cases have been reported in the literature. Here, we present a unique case of successful management of a concurrent case of thyrotropin-prolactinoma with papillary thyroid carcinoma. *Case Presentation*. A 50-year-old Moroccan woman underwent a total thyroidectomy and complementary totalization by iratherapy for papillary thyroid carcinoma, who presented persistence of an inappropriate secretion of the thyroid-stimulating hormone (TSH > 4 mUI/L) despite of levothyroxine suppressive therapy (300 *μ*g/d). After eliminating noncompliance, interfering medicines, and thyroid malabsorption, a pituitary adenoma (12 mm) was documented at magnetic resonance imaging. The patient has had transsphenoidal pituitary adenomectomy with histology confirming a thyrotropin-prolactin-secreting adenoma. After surgery and lanreotide treatment failures, we noted a complete response (TSH < 0.5) with cabergoline treatment (3 mg/week).

**Conclusion:**

The unusual association of thyroid adenocarcinoma and TSHoma enriches the hypothesis of a potential link between thyrotropic hypersecretion and thyroid carcinogenesis. Our case also illustrates the difficulty of monitoring thyroid carcinoma in nonremission of a TSHoma.

## 1. Introduction

The thyrotropin-secreting adenomas (TSHomas) are very rare; their prevalence accounts for 0.5–3% of all pituitary tumors [1]. The sensitive laboratory assays and the accuracy of magnetic resonance imaging currently allow early and precise diagnosis. Simultaneous coexistence of TSHoma and papillary thyroid carcinoma (PTC) is still exceptional, and so far, only sixteen cases have been reported [2–14]. This association suggests the involvement of TSH in thyroid carcinogenesis and raises the difficulty of monitoring levothyroxine suppressive therapy in thyroid carcinoma after a total thyroidectomy.

## 2. Observation

**Figure 1:**
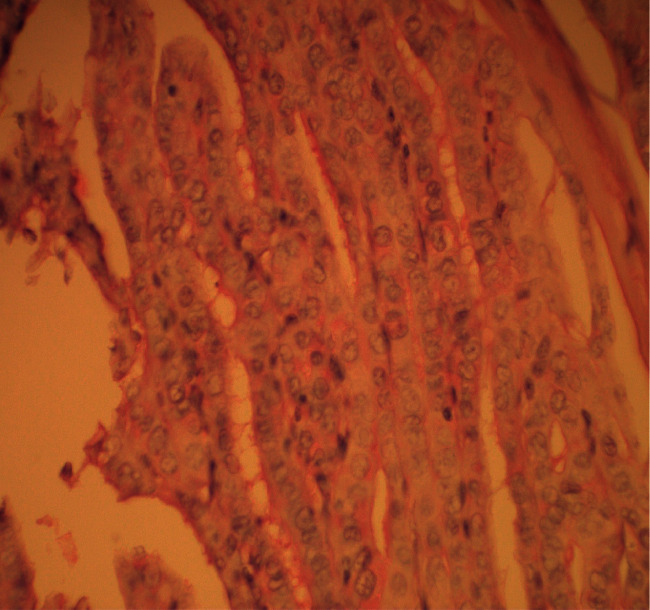
Nuclear features of papillary thyroid carcinoma, ground glass appearance, intranuclear grooves, nuclear crowding, and overlapping (x400).

**Figure 2:**
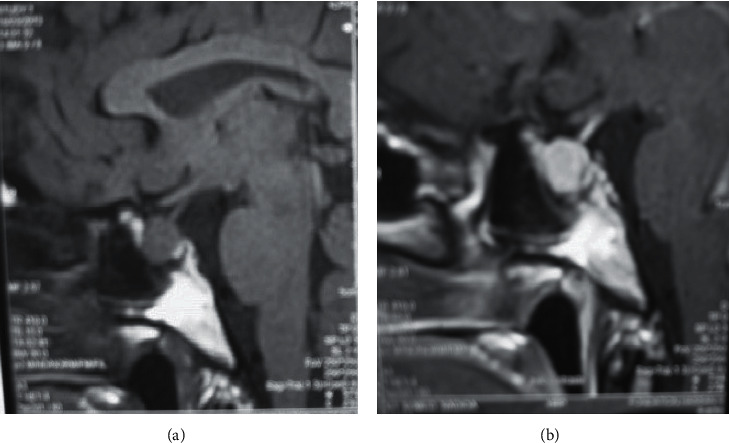
Preoperative pituitary MR images (T1-weighted, contrast, and sagittal view) showing (a) pituitary macroadenoma 12 mm without mass effect on adjacent structures (especially optic chiasm) and (b) strong homogeneous enhancement of the pituitary adenoma after contrast administration.

A 50-year-old Moroccan woman, followed for type 2 diabetes, who underwent a total thyroidectomy with histology confirming papillary thyroid carcinoma (measuring 10 mm) classified T1b Nx M0 (Figure 1). She also received an adjuvant therapy with 100 mCi of radioactive iodine. Posttherapy whole-body scanning revealed fixation in the right thyroid bed compatible with thyroid remnant without metastasis. Cervical ultrasound did not objectify any residue or lymphadenopathy. We noted persistently unrepressed TSH after gradual dose levothyroxine suppressive therapy up to 300 *μ*g per day (4 *μ*g/kg/day); her serum TSH level fluctuating from 4.5 to 50 *μ*IU/mL and thyroglobulin (Tg) level was 6.8 ng/mL. We had quickly eliminated noncompliance, interfering medicines, and thyroid malabsorption, and we suspected a syndrome of inappropriate TSH secretion (SITSH). A pituitary MRI showed a macroadenoma in the sella turcica (10 × 12 mm), without local mass effect on adjacent structures, especially optic chiasm (Figure 2). The specific investigation was in favor of TSHoma, in particular the absence of TSH suppression in the T3 suppression test (100 *μ*g/day divided in 3 administrations), the serum alpha-subunit levels (*α*-GSU) was 4.9 ng/mL (normal postmenopausal value 0.6 to 1.5 ng/mL), and *α*-GSU/TSH molar ratio was 1.4 (normal <1). Other investigations to evaluate pituitary function were normal (Table 1).

**Figure 3:**
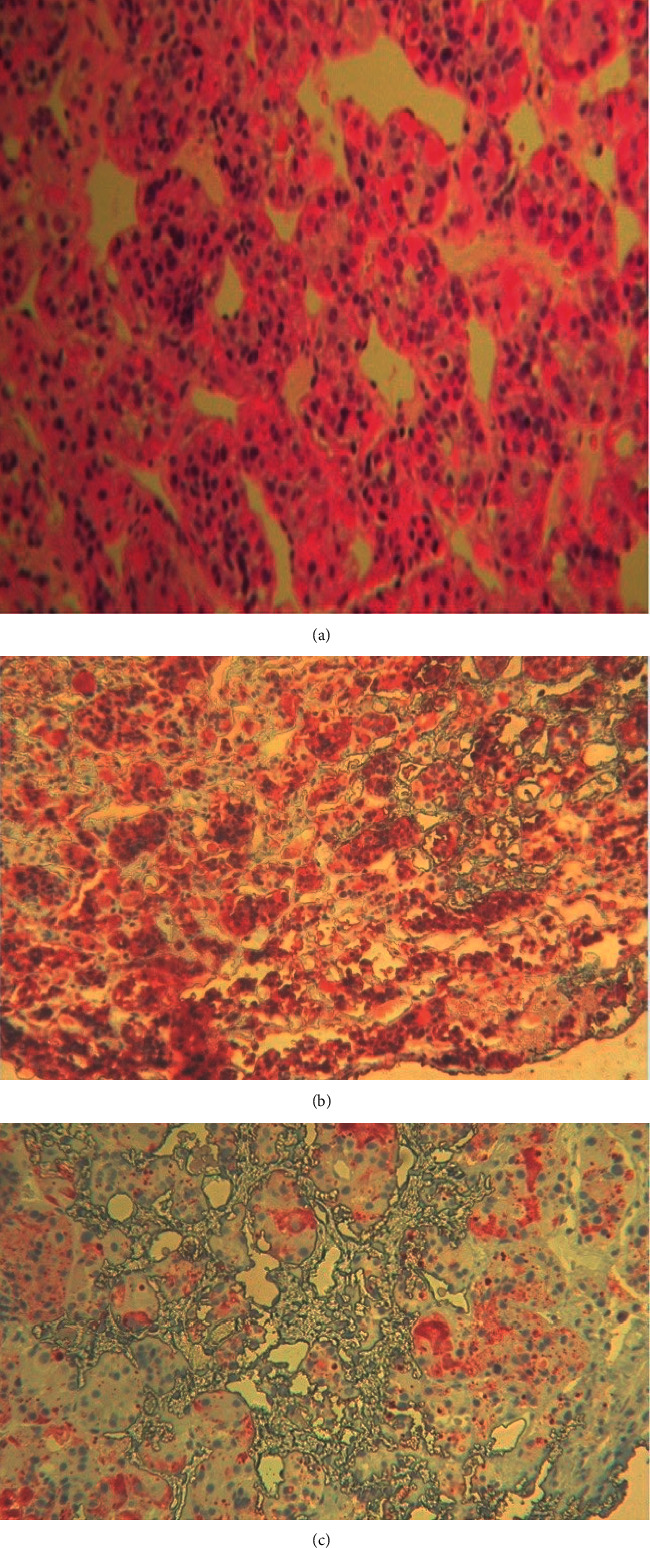
Histopathological and immunohistochemical study showing (a) histopathological features of pituitary adenoma (x200), (b) 50% of ßTSH immunoreactivity (x400), and (c) 25% of prolactin immunoreactivity (x400).

Accordingly, the patient has had transsphenoidal pituitary adenomectomy with histology confirming a thyrotropin-prolactin-secreting adenoma (50% of *β*TSH immunoreactivity and 25% of prolactin immunoreactivity) (Figure 3).

Three months after surgery, no residual tumor was apparent on pituitary MRI, but we noted a persistence of inappropriate secretion of TSH under 300 *μ*g/d of levothyroxine, TSH level was 20 *μ*IU/mL, FT4 level was 22.4 pmol/l, FT3 level was 5.6 pmol/l, and Tg level was 22.7 ng/mL (Table 1). Given the absence of biological remission after surgery, the patient underwent lanreotide therapy at 90 mg per month for three months, but the TSH level was still increased which provokes the stop of lanreotide. The TSH level was lowered for the first time after switching to cabergoline therapy and it allowed a complete response under 3 mg per week (Table 1). The follow-up is eight years after cabergoline therapy with a good cardiac tolerance: the patient has managed to achieve complete remission of thyroid cancer without any biochemical or structural recurrence (low nonstimulated thyroglobulin levels, normal cervical ultrasound, and negative 131I-whole-body scan). Automatically levothyroxine dosage was gradually reduced to 125 *μ*g/day.

## 3. Discussion

We present here a case of thyroid cancer in coexistence with thyrotropin-prolactin-secreting adenoma. The sixteen published cases are reviewed and compared with our patient at the level of many parameters: age, sex, size of thyroid cancer, size of TSHoma, immunohistochemical study results, therapies used, and observed evolution (Table 2) [2–14].

TSH-suppressive hormonal therapy is a cornerstone of thyroid carcinoma therapy. Unrepressed TSH after levothyroxine suppressive therapy first evokes noncompliance, interfering medicines, thyroid malabsorption, and biological interference, before conducting further investigations for SITSH. These diagnoses can be easily excluded by questioning, monitoring of drug intake, testing malabsorption, and repeating serum TSH measurement with several laboratory methods to exclude transitory changes and biological interference [15, 16]. Once these diagnoses are excluded, we are therefore faced with SITSH and the second step is to differentiate between TSHoma and syndromes of thyroid hormone resistance (RTH) [16, 17]. The differential diagnosis is complicated, so clinical presentations, laboratory assessment, and imaging advances may help and guide diagnosis (Table 3). T3 suppression test is the most specific and sensitive test, especially in patients with thyroid ablation. *α*-GSU may also be used; an elevated *α*-GSU concentration or a high *α*-GSU/TSH molar ratio favors the diagnosis of TSHoma in particular, in macroadenoma; these parameters are within the normal range in the case of a microadenoma [18]. Due to their low sensitivity and high variability in several conditions such as age and menopause, *α*-GSU and GSU/TSH molar ratio may rarely help in the differential diagnosis [19]. Other tests are useful in the differential diagnosis like the increase of liver parameter such as the sex hormone binding globulin (SHBG) in the case of TSHomas, while it is normal in RTH [20, 21] (Table 3). This test was not performed in our patient because she was under a supraphysiological dose of levothyroxine. In addition, a difficulty of imaging lies in the presence of pituitary incidentalomas in 10–20% of the normal population and they are more frequent than TSHoma, so autonomous TSH production must be established [22, 23]. Finally, an association between TSHoma and RTH has been reported, hence the interest in gathering clinical, hormonal, and morphologic information [24]. In our patient, the radiological findings (pituitary macroadenoma) were associated with clinical data (no family history of thyroid disease) and biological findings (unrepressed TSH in T3 suppression test and elevated *α*-GSU level and *α*-GSU/TSH molar ratio), which allowed diagnosis of TSHoma.

Many cases of TSHomas are completely asymptomatic which explains why the majority of TSHomas are macroadenomas as in our patient [20] Therefore, it is important that the clinician be alerted to persistently unrepressed TSH after levothyroxine suppressive therapy in order to be able to carry out investigations and treat TSHoma as early as possible. On the other hand, it is so difficult to control and to monitor the carcinologic side of PTC when it coexists simultaneously with TSHoma as in our case.

The unusual association of PTC with TSHomas enriches the hypothesis of a potential link between thyrotropic hypersecretion and thyroid carcinogenesis. Thus, the role of TSH in carcinogenesis and development of PTC have been suggested in several studies where we noted high incidence of PTC in the patients with TSHomas and a meta-analysis of 28 studies have objectified a significant association between TSH levels and the risk of PTC [20, 25, 26]. Conversely, thyroidectomy when performed first before resection of pituitary adenoma may affect growth rate and secretion of the TSHoma due to diminished negative feedback effect of thyroid hormones [9]. This is the same mechanism described in Nelson's syndrome after adrenalectomy in Cushing's disease.

There are several therapeutic modalities for TSHoma, but the first line therapy is transsphenoidal pituitary surgery in order to remove tumor mass and to normalize TSH, with a success rate of 80% [27, 28]. In our case, surgery allowed removing tumor mass without TSH suppression. Second-line treatments include repeat pituitary surgery, radiation therapy, and medical therapy; they should be considered after failed transsphenoidal surgery and when the patient is not amenable to surgery or he declines it. Radiotherapy (conventional fractional radiotherapy or radiosurgery) may also be indicated in the treatment of TSHoma; however, the benign character of the condition, the potential side effects of radiation in particular hypopituitarism, and the long delay between radiation and efficacy will make its use less frequent, especially when medical therapies provide good control of the disease as we will see below [29]. Somatostatin analogs (SA) are the best choice of medical alternative treatment; they restore normal thyroid function in about 95% and shrink the size of TSHoma in up to 50% of patients [30–32]. Resistance to SA in our case is most likely related to nonexpression of somatostatin receptors. The presence of dopamine receptors on the thyrotroph cells justifies the therapeutic use of dopaminergic agents in TSHomas; however, efficiency in control of TSH secretion and tumor shrinkage was lower compared to SA and the best results were obtained in mixed TSH/prolactin adenomas [30, 31, 33–36]. The immunohistochemical study had objectified an immunofixation for TSH and also for prolactin with the absence of clinical or biological symptoms of prolactinoma on admission (neither amenorrhea nor galactorrhea and normal serum prolactin); this situation defines the true silent prolactinoma. It is a rare entity, especially in macroprolactinoma, most frequently associated with somatotropic adenomas. Discovery of PRL positive staining may provide an option for dopamine agonists in the treatment of recurrent silent prolactinomas as illustrated in our observation [37].

## 4. Conclusion

The unusual association of PTC with TSHomas enriches the hypothesis of a potential link between thyrotropic hypersecretion and thyroid carcinogenesis. Our observation highlights efficiency of cabergoline in control of TSH secretion, especially in the presence of mixed TSH/prolactin adenomas, without forgetting the difficulty encountered to control and to monitor the carcinologic side of PTC when it coexists simultaneously with TSHoma.

## Figures and Tables

**Table 1:** Pertinent laboratory findings.

Investigations	Before admission	At admission	After surgery	After lanreotide	After cabergoline	Reference range
TSH (*μ*IU/mL)	4.5–50	35	20	6	0.5	0.35–4.95
FT4 (pmol/L)	—	23	24	22	19	12–22
FT3 (pmol/L)	—	5.3	6.7	6.6	5.9	2.8–7.1
Thyroglobulin (ng/mL)	—	6.8	22.7	8	0.4	<1
*α* subunit (ng/mL)	—	4.9	—	—	—	0.6–1.5
*α*-GSU/TSH molar ratio	—	1.4	—	—	—	<1
LH (IU/L)	—	36.8	—	—	—	2.4–12.6
FSH (IU/L)	—	84.1	—	—	—	3.5–12.5
Estradiol (pmol/L)	—	51	—	—	—	46–607
Prolactin (mIU/L)	—	250	230	—	90	72–511
Cortisol (nmol/L)	—	525	—	—	—	171–536
ACTH (pg/mL)	—	40	—	—	—	7–63
IGF1 (*μ*g/L)	—	102	—	—	—	93–245

**Table 2:** Literature review of thyroid cancer with TSHoma.

Case	Age	Sex	Size of TC (mm)	Size of PA (mm)	IHC of PA	Therapies of TSHoma	Therapies of TC	Evolution of TSHoma	Evolution of TC	References (year of publication)
1	55	M	50	30	TSH	TS adenomectomy	Total thyroidectomy/iratherapy	Remission	Remission	[2] (1991)
2	37	F	20	10	TSH	Refusing treatment	Total thyroidectomy/iratherapy	NR	Remission	[3] (1998)
3	27	F	30	10	TSH	Octreotide/TS adenomectomy	Partial thyroidectomy	Remission	NR	[4] (2000)
4	45	F	20	15	TSH	Octreotide/TS adenomectomy	Total thyroidectomy	Remission	NR	[5] (2001)
5	47	F	8	4	TSH/PRL	TS adenomectomy	Total thyroidectomy/iratherapy	NR	Remission	[6] (2006)
6	50	M	17	3	TSH	Refusing treatment	Total thyroidectomy	Stability	Remission	[7] (2009)
7	57	F	8	26	TSH/GH	Octreotide	Total thyroidectomy/iratherapy	Remission	Remission	[8] (2010)
8	38	F	40	14	TSH/FSH	Octreotide/TS/radiosurgery	Total thyroidectomy/iratherapy	Stability	Remission	[9] (2013)
9	27	F	10	28	TSH	TS adenomectomy/lanreotide	Total thyroidectomy/iratherapy	Partial response	Remission	[9] (2013)
10	33	F	14	20	TSH/GH	TS adenomectomy/cabergoline	Total thyroidectomy/iratherapy	Remission	Remission	[10] (2014)
11	47	M	15	19	TSH	TS adenomectomy/lanreotide	Total thyroidectomy/iratherapy	Remission	Remission	[11] (2015)
12	46	M	12	7	TSH/GH	TS adenomectomy/lanreotide	Total thyroidectomy/iratherapy	Remission	Remission	[11] (2015)
13	42	M	40	12	TSH	TS adenomectomy	Total thyroidectomy/iratherapy	Remission	Remission	[11] (2015)
14	44	F	30	16	TSH/GH/FSH	Octreotide/TS adenomectomy	Total thyroidectomy/iratherapy	Remission	Remission	[12] (2017)
15	27	F	NR	NR	TSH	Surgical resection for nasopharyngeal tumor (ectopic TSHoma)	Total thyroidectomy/iratherapy	Remission	Remission	[13] (2017)
16	57	M	NR	30	TSH	Octreotide/TS adenomectomy	Partial thyroidectomy	Remission	Remission	[14] (2018)
Our case	50	F	10	12	TSH/PRL	TS adenomectomy/lanreotide/cabergoline	Total thyroidectomy/iratherapy	Remission	Remission	-(2021)

NR, not reported; TC, thyroid cancer; PA, pituitary adenoma; IHC, immunohistochemistry; TSH, thyrotropin; PRL, prolactin; GH, growth hormone; FSH, follicle-stimulating hormone; TS, transsphenoidal.

**Table 3:** The differential diagnosis markers between TSH-secreting adenomas (TSHomas) and resistance to thyroid hormones (RTH).

	TSHoma	RTH
Family history of thyroid disease	Absent	85%
Unsuppressed TSH or increased	Present	Present
Elevated thyroid hormone levels	Present	Present
Nycthemeral profile of TSH	Absent	Present
High levels of SHBG (TeBG)	Present	Absent
Increased *α* subunit	65%	3%
*α*-GSU/TSH molar ratio	>1%	<1%
Increase in TSH after a TRH	Negative	Positive
T3 suppression test	No TSH suppression	Suppression of TSH
Somatostatin test	FT4 ↓ >30%	FT4 is not affected
Multihormonal production	Possible	Absent
MRI pituitary	98%	10–20%
DNA mutation analysis	−	+
